# Living in a Multi-Risk Chaotic Condition: Pandemic, Natural Hazards and Complex Emergencies

**DOI:** 10.3390/ijerph17165635

**Published:** 2020-08-05

**Authors:** Mohammad Amin Hariri-Ardebili

**Affiliations:** 1 College of Engineering and Applied Science, University of Colorado, Boulder, CO 80309, USA; mohammad.haririardebili@colorado.edu; Tel.: +1-303-990-2451; 2 College of Computer, Mathematical and Natural Sciences, University of Maryland, College Park, MD 20742, USA

**Keywords:** multi-risk, COVID-19, pandemic, natural hazard, protest, healthcare system

## Abstract

Humans are living in an uncertain world, with daily risks confronting them from various low to high hazard events, and the COVID-19 pandemic has created its own set of unique risks. Not only has it caused a significant number of fatalities, but in combination with other hazard sources, it may pose a considerably higher multi-risk. In this paper, three hazardous events are studied through the lens of a concurring pandemic. Several low-probability high-risk scenarios are developed by the combination of a pandemic situation with a natural hazard (e.g., earthquakes or floods) or a complex emergency situation (e.g., mass protests or military movements). The hybrid impacts of these multi-hazard situations are then qualitatively studied on the healthcare systems, and their functionality loss. The paper also discusses the impact of pandemic’s (long-term) temporal effects on the type and recovery duration from these adverse events. Finally, the concept of escape from a hazard, evacuation, sheltering and their potential conflict during a pandemic and a natural hazard is briefly reviewed. The findings show the cascading effects of these multi-hazard scenarios, which are unseen nearly in all risk legislation. This paper is an attempt to urge funding agencies to provide additional grants for multi-hazard risk research.

## 1. Background

Human existence involves exposure to many hazards [1], and various low to high risk scenarios. While understanding a hazard and its associated risks may help prevent or reduce adverse consequences, in many instances, people are unaware of the risks involved, making it difficult to fight against an invisible enemy. Risk aversion is a robust characteristic of human decision making, meaning people are less likely to gamble on something when they are unsure if they will obtain the desired outcome. However, planning for risks becomes even more challenging when considering that we live in a multi-risk world with infinite natural and man-made hazards, many of which we cannot control and can occur at any time.

**Figure 1:**
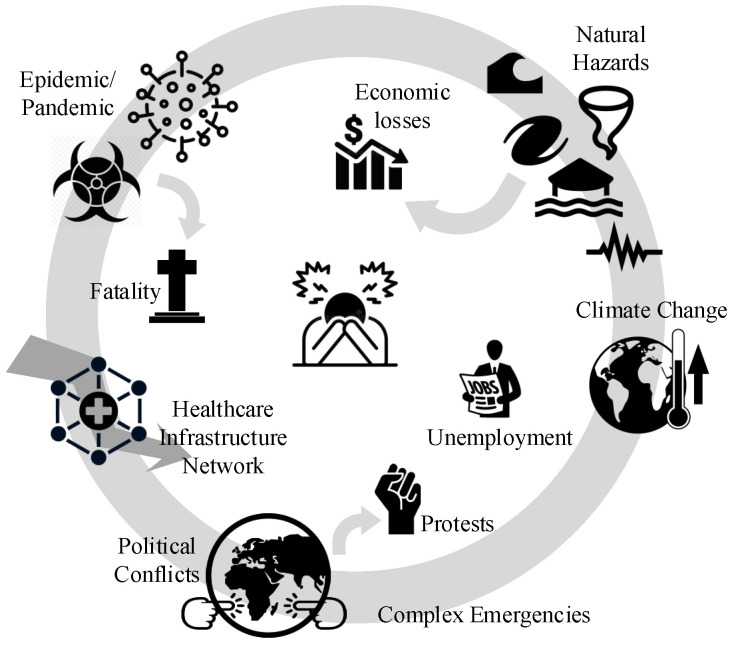
A multi-risk condition.

The year 2020 will be remembered in the U.S. for several reasons: (1) The coronavirus began to spread throughout the nation starting in February, with the first confirmed case reported on 21 January, according to CDC (https://www.cdc.gov/mmwr/volumes/69/wr/mm6918e2.htm). (2) There were multiple natural hazards (NH) around the country, including two devastating dam failures in Michigan and over 500 earthquakes in western Nevada (https://www.sfgate.com/earthquakes/article/Nevada-Tonopah-earthquakes-6-5-aftershocks-15283968.php), and an above-normal Atlantic hurricane season expected (https://www.cnn.com/2020/05/11/us/2020-atlantic-hurricane-season-fast-facts/index.html). (3) Complex emergency (CE) situations arose at both national (e.g., Black Lives Matter protests) and international (e.g., U.S.–China and U.S.–WHO tensions) levels. Other notable conditions included the ongoing climate crisis and economic contractions due to nation-wide stay-at-home orders. For example, GDP fell 4.8% in Q1 2020, and unemployment increased by more than 10% between April and May. Figure 1 illustrates the combination of multi-hazard factors that many citizens are facing.

This paper tries to briefly review three distinct risk sources that the U.S. is facing, Section 2; followed by the concept of multi-risk analysis in Section 3. A perspective on low- to high-probability risk scenarios for healthcare system impacts, functionality loss, and recovery duration is provided in Section 4. Finally, the concept of NH-induced evacuation and sheltering during pandemic conditions is revisited in Section 5.

## 2. Single-Hazard Risk

### 2.1. COVID-19 Pandemic

In December 2019, the novel coronavirus pandemic, known as COVID-19, emerged from Wuhan, China [2]. It is the most recent biological hazard and has resulted in a global outbreak. COVID-19 is a potential zoonotic disease with a low to moderate mortality rate. Person-to-person transmission may occur through droplets or direct contact [3], and therefore isolation of cases and contact tracing are essential to controlling COVID-19 outbreaks, though the probability of successfully controlling an outbreak decreases as the number of initial cases increases [4].

As of 4 August 2020, there are about 4.8 million confirmed cases, 2.3 million recovered patients, and about 160,000 deaths related to COVID-19 in the U.S. alone [5]. Multiple researchers have studied the risks associated with a COVID-19 outbreak [6,7]. Although most of these studies are in preliminary stages, different types of forecasting models have been proposed to predict the temporal and spatial distributions of the virus when subjected to various constraints [8,9,10]. These projection models account for factors such as the behaviors of citizens, impacts of social distancing, effectiveness of face coverings, consequences of reopening, capacity of the healthcare system, pre-existing health conditions and age groups [11,12]. It is noteworthy that there is an epistemic uncertainty (lack of current knowledge) [13] in the exact number of infections and their spatial distribution. Therefore, making any decision about the reopening of the states/cities is very difficult and challenging. This already caused extra political problems and legal challenges. According to CNN (https://www.cnn.com/2020/07/16/politics/georgia-kemp-mask-mandate/index.html), Georgia governor, Brian Kemp, announced that he is suing Atlanta Mayor, Keisha Lance Bottoms, over the city’s mask mandate, claiming the measure violates his emergency orders.

**Figure 2:**
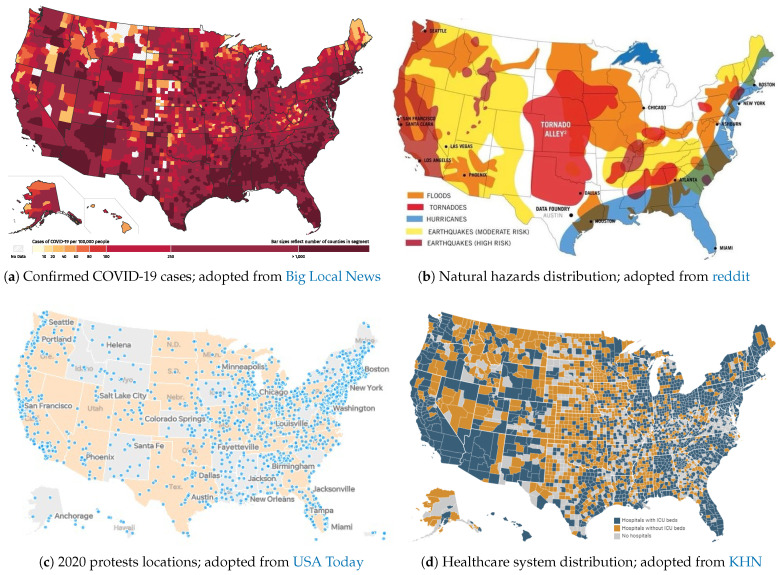
Spatial distribution of risk of exposure (pandemic), potential natural hazard, and mass gathering (of protests) across the U.S.; all maps are approximate and for illustration purposes only.

The risk of exposure to COVID-19 is an important factor in subsequent life loss (LL) estimations. Among other factors, it is highly dependent on location, human concentration, and safety protocols. An approximate COVID-19 risk map is shown in Figure 2a (as of 18 July 2020). The map is based on incidence rate (i.e., the number of confirmed cases per 100,000 people), and since it accounts for population density, it offers a more reliable metric for exposure risk. The map is subjected to temporal changes.

### 2.2. Natural Hazards

Natural hazards are the result of a series of natural processes that have operated throughout earth’s history [14]. Hazard analysis refers to a process of recognizing hazards that may arise from a system or its environment, documenting their unwanted consequences, and analyzing their potential causes [15]. Natural hazards are classified as geophysical (e.g., earthquake, volcanic activity), meteorological (e.g., tornado), hydrological (e.g., flood), climatological (e.g., drought), biological (e.g., epidemic), and extraterrestrial (e.g., impact).

Since the start of the COVID-19 pandemic in January 2020, several natural disasters have been reported, including: (1) dozens of tornados in southern states in the U.S. between 12 and 13 April (36 fatalities); (2) A 6.5 magnitude earthquake struck the western area of Nevada on 15 May, damaging the main highway; (3) two dam breaks on 19 May in Michigan, U.S. (with 11,000 evacuees); (4) tropical Storm Cristobal made landfall on 7 June, near the mouth of the Mississippi River and the island of Grand Isle in Louisiana, brought winds of up to 85 km/h, and spawned a tornado in Florida; (5) seven inches of rain caused flash floods in Wisconsin, washed out roads, and declared a state of emergency on 29 June; and (6) as of late July, Hurricane Hanna has roared ashore onto the Texas Gulf Coast as a Category 1 storm.

In the U.S., FEMA provides the major disaster declarations every year [https://www.fema.gov/disasters/year/2020?field_dv2_declaration_type_value=All]. Figure 2b is one of many maps showing the approximate locations of various natural hazards across the country. The majority of risk maps for natural disasters are developed based on their economic impact to properties (e.g., loss).

### 2.3. Complex Emergencies

While multiple complex emergencies have developed since the start of the pandemic (e.g., U.S.-China tensions, U.S. military movements in the Middle East), only those directly affected by the spreading of COVID-19 inside U.S. borders are discussed in this paper. These can be categorized into two main groups: (1) normal condition actions and (2) emergency condition responses. Both categories are somewhat related to mass gatherings, which pose significant public health challenges to health care professionals and governments [16]. Historically, sporting, religious, music, and other mass gatherings have enabled the global spread of infectious diseases [17]; the situation can become worse when face coverings, social distancing, and other preventative actions are not fully observed by attendees. Authorities in each community must try to flatten the transmission curve to give scientists more time to find a cure; however, mass gatherings move the needle in the opposite direction.

Multiple researchers have shown that the perceived risk of COVID-19 is affected by politically-motivated interpretations of the risk. These patterns persist even in the face of state-level mandates to close schools and non-essential businesses [18]. Studies show that political partisanship may play a role in determining perceived risk during a pandemic, with potentially significant changes in public health outcomes. According to Painter and Qiu [19], residents in Republican counties are less likely to comply with stay-at-home orders than those in Democratic counties. Similarly, Democratic-leaning counties responded more to recommendations from Republican governors than from Democratic ones [20]. According to Adolph et al. [21], the results of the state-level database analysis for five social distancing policies across all fifty states revealed that: all else equal, Republican governors and governors from states with more President Trump supporters were slower to adopt social distancing policies. Furthermore, it is reported that U.S. counties with lower per capita income were associated with significantly reduced social distancing mandates [22]. Thus, the geographical location of a pandemic is an important factor in its spread. According to Dincer and Gillanders [23], in communities where corruption is endemic, observing social distancing during sheltering and implementation of mitigation strategies is difficult. We should also highlight the mutual trust between individuals and their communities [24], which yields a successful emergency mission during a pandemic outbreak.

The 2020 U.S. presidential election rallies are also a hot topic amongst voters. While many believe the rallies should stop, President Trump did not cancel his June campaign in Tulsa, Oklahoma. According to CNN (https://www.cnn.com/2020/06/25/politics/trump-tulsa-rally-coronavirus/index.html), following this rally (20 June), at least eight staffers who were part of the rally preparation tested positive, and the rest who attended the rally were quarantined. One may note that the incubation period for COVID-19 is about 14 days, but the CDC (https://www.cdc.gov/coronavirus/2019-ncov/hcp/clinical-guidance-management-patients.html) announced the median time of 4–5 days (other resources reported similar data [25]). To date, there is no scientific research showing the direct correlation between rallies and transmission of COVID-19. However, according to Fox News (https://www.foxnews.com/politics/trump-campaign-says-two-more-staffers-who-attended-tulsa-rally-tested-positive-for-coronavirus), *at the same time as the rally, Tulsa County was experiencing its own spike in infections, which drew concerns that Trump’s indoor rally could be a “super spreader” event for the virus.*

**Figure 3:**
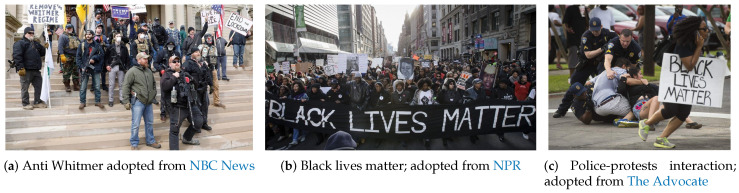
Complex emergencies and protesting during COVID-19.

One form of mass gathering during the pandemic has been protesting or marching by different civil rights groups, and the U.S. has seen several such protests over the past three months. On 24 April, nearly 1500 people gathered at the Wisconsin State Capitol in Madison to protest. Two weeks later, the Wisconsin Department of Health Services confirmed 1986 cases of COVID-19. Of those, 72 people reported having attended a large gathering, though patients were not asked specifically if they had attended the protest [26]. It was observed that many protesters did not maintain a six-foot distance from others or wear masks. On 15 April, there was a protest in Lansing, Michigan against the state’s governor, Gretchen Whitmer, and her COVID-19 lockdown, Figure 3a. Again, there was no sign that people were taking the COVID-19 related public health advice seriously.

Following George Floyd’s death on June 1 by a white police officer in Minnesota, a new wave of Black Lives Matter protests began in the U.S. While many worried about catching COVID-19 during these marches [27], they decided to take the risk anyway, Figure 3b. While some of the protesters tried to follow public health advice (i.e., wearing masks, distancing, using hand sanitizer, and getting tested for COVID-19), but there is no perfectly safe way to demonstrate in large groups during a pandemic. Concerns about racism and discrimination have also arisen during the COVID-19 outbreak [28]. COVID-19 policy responses have disproportionately affected people of color and immigrants-people who are over-represented in lower socioeconomic groups, have limited access to healthcare, and work in precarious jobs [28]. The question for many was: “Which is worse: protesting with an increased short-term risk of fatalities due to COVID-19 or staying at home and enduring sustained systematic racism?”

For many black people and their allies, the risks associated with protesting did not outweigh the risks of doing nothing, which some equated with “one in every 1000 black men dying at the hands of police”. According to The Guardian (https://www.theguardian.com/us-news/2020/jun/03/protests-police-covid-19-coronavirus-spread), it is impossible to know how many people at these marches were asymptomatic carriers, and that is really scary. Protests, like those mentioned above, are now taking place nation-wide; Figure 2c maps the locations of protests in the U.S. related to George Floyd’s death and the larger Black Lives Matter movement.

Finally, the scenario was worsened by police tactics used to subdue protesters, Figure 3c. According to Wired (https://www.wired.com/story/police-tactics-could-turn-protests-into-covid-19-hot-spots/), some police tactics could turn protests into COVID-19 hot spots. While large crowds already carry a risk of transmission, the situation is exacerbated when police deploy tear gas against protesters, causing them to cough on each other (spraying virus-laden droplets into the environment), or bus them to jails in groups. Tear gas and pepper spray make it nearly impossible to breathe while wearing a mask, and mass arrests or detainments are very risky, not just for the people arrested but also for the jail staff, the court staff, and their families. In any case, the police have to respond to vandalism and theft (in any form) in which people try to damage public properties, steal from stores, and alter the peaceful protest. Also, the police should confront anarchist agitators, and criminal opportunism amid the chaos. It is important to note, therefore, that both protesters and police face significant risks. According to the Military (https://www.military.com/daily-news/2020/06/10/national-guard-covid-19-diagnoses-after-protests-are-disturbing-sign-fauci-says.html), an undisclosed number of the roughly 1200 D.C. Guard members sent to respond to the protests now have COVID-19. Two members of the Nebraska National Guard also tested positive.

The readers should note that all the statements (especially the political ones) throughout this paper do not reflect the personal political view point of the author in any way. All the statements are carefully selected from peer-reviewed documents and those reported by the news. None of the statements aim to defend a political party and/or opinion, but to bring to the attention of readers, the complex situation we are living.

### 2.4. Healthcare Availability

Finally, all the above-mentioned hazard sources (and their combined effects) should be studied in the context of the healthcare system performance, See Section 4. A better healthcare system may reduce the devastating consequences of large-scale fatalities. According to KHN (https://khn.org/news/as-coronavirus-spreads-widely-millions-of-older-americans-live-in-counties-with-no-icu-beds/), more than half of counties in the U.S. have no hospital ICU beds, which poses a particular danger for more than seven million 60+ years old people facing the spread of COVID-19. They released a map (based on 2018 and 2019 reported data), showing the counties with and without hospitals, and counties that do have ICU beds, Figure 2d. As can be seen, there is considerable heterogeneity in the distribution of the ICU beds (with some having just one bed available for thousands of senior residents). One may normalize the above-discussed multi-hazard sources with respect to the available healthcare system in each county. In this way, the impact of the healthcare system (as a secondary hazard source, in case it is not sufficient) is incorporated in overall risk calculation.

## 3. Multi-Hazard Risk

Each of the individual risk factors explained in Section 2 can be catastrophic and devastating if the individuals and/or society/community are not prepared already [29]. However, the critical question is “Are we ready for combination of these risks?”. Therefore, we need to talk about the framework of multi-risk analysis, keeping in mind the fundamental differences between hazard and risk:

Risk ∼ Hazard × Impact on asset × Consequences of impact; Ref.[30](1)

Risk ∼ Hazard × Value at risk × Vulnerability; Ref.[31](2)

The concept of multi-risk analysis is well established in natural hazards [30,31]. European Commission [32] defines the multi-hazard assessment as:

“*to determine the probability of occurrence of different hazards either occurring at the same time or shortly following each other, because they are dependent from one another or because they are caused by the same triggering event or hazard, or merely threatening the same elements at risk without chronological coincidence.*”

Therefore, a multi-hazard assessment can be studied from two perspectives [33]:

- Independent hazards threatening a given area: the main concern in this effort is harmonization of the hazard assessment, meaning that all should have a similar basis [31].
- Hazard interactions, triggering or cascade effects: in this effort, the occurrence of one event could affect the probability of occurrence in others (usually accelerates them). Once this concept is propagated into a chain of events, the Bayesian networks framework can be used [34]. For two events with occurrence of $e_{1}$ and $e_{2}$, the probability of $e_{1}$ occurrence, $P[e_{1}]$, is:
  (3) $$P\left[e_{1}\right]=P\left[e_{1}|e_{2}\right].P\left[e_{2}\right]+P\left[e_{1}|{\bar{e}}_{2}\right].P\left[{\bar{e}}_{2}\right]$$
  where the bar sign presents the non-occurrence condition. This equation can be generalized for *N* events.

**Figure 4:**
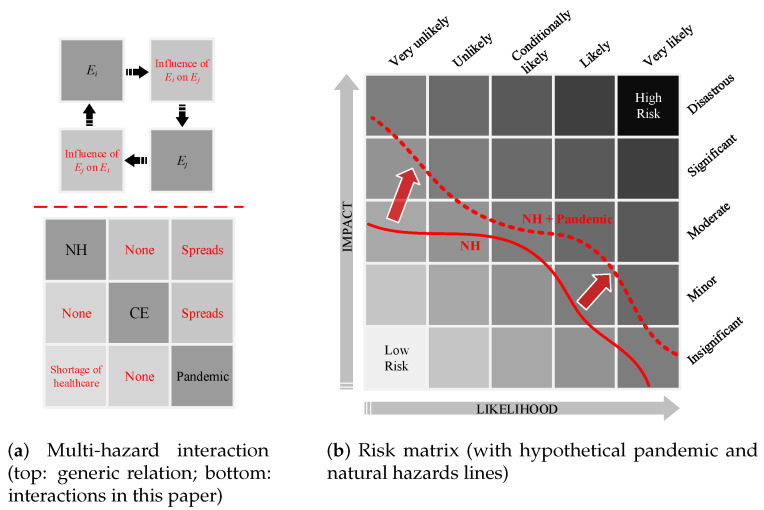
Matrix presentation of multi-hazard and multi-risk.

Liu et al. [34] proposed a simple matrix approach to identifying the interactions between various hazards. According to Figure 4a (upper part), the interaction of any two hazards $E_{i}$ and $E_{j}$ can be determined by understanding their impacts on one another. For three hazard sources in this paper, such interactions are assessed in Figure 4a (lower part). While the single-hazard sources take the diagonal cells, their clockwise influence/interaction fill out the off-diagonal cells. For example, this matrix shows that occurrence of a natural hazard may spread the pandemic, while an ongoing pandemic does not change the probability of occurrence of a natural hazard. Each of these major hazard sources also has various sub-categories. For example, natural disasters include earthquakes, floods, and hurricanes, while pandemics and epidemics include outbreaks of COVID-19 or an intensified seasonal influenza.

A combination of all single-hazard sub-categories and multi-hazard scenarios can be illustrated on a risk matrix, Figure 4b. A risk matrix is a simple way to present the severity and probability of various events [35], increasing the visibility of risks to assist with decision making. Assuming the evaluation metric is the number of fatalities, a path can be developed connecting all the single natural hazards [36]. The same approach can be followed to add in the effects of a pandemic. Presumably, both the likelihood of fatalities and their impacts are increased (or may stay constant in some instances) when a natural hazard occurs during a pandemic. This can be expanded for any combination of two or three hazard sources (not shown in this figure).

Since this paper focuses on life loss (i.e., human fatalities) as a main metric for risk analysis, it is important to distinguish the differences between individual and societal risk measures. The individual risk, R${}_{I}$, is defined as the probability that an average unprotected person, permanently present at a certain location, is killed due to a hazardous event [1]:

(4) $$R_{I}=P\left[e_{i}\right].P\left[LL|e_{i}\right]$$

where $P[e_{i}]$ is the probability of hazardous event *i*, and $P[LL|e_{i}]$ is the probability of life loss due to *i*th hazardous event.

On the other hand, the societal risk (the expected loss), R${}_{S}$, can be approximated as:

(5) $$R_{S}={\;\int \int }_{A}R_{I}\left(x,\;y\right).m\left(x,\;y\right)dxdy$$

where m(x, y) is the population density at location (x, y), and *A* is the area.

## 4. Multi-Hazard on Healthcare System

Arguably one of the most important tasks in any community is keeping the healthcare system as resilient as possible. Resilience refers to the capacity of a system, community, or society to adapt to potential hazards by resisting or changing in order to reach and maintain an acceptable level of functionality and structure [37,38]. The concept of resilience was first introduced in the field of psychology [39] and has been rapidly adopted by environmental [40] and social sciences [41]. Individual researchers have addressed the resilience of communities against natural hazards (e.g., climate-resilient [42], earthquake-resilient [43], flood-resilient [44], pandemic-resilient [45], and politically-resilient [46]).

The concept of resilience has risen in popularity during the COVID-19 pandemic, prompting many researchers from various fields to re-evaluate their protocols, systems, and communities to understand how they could recover from adverse effects of COVID-19. Among hundreds of publications, the most notable have focused on medical resilience [47,48], mental resilience [49,50,51], tourist resilience [52], food system resilience [53], supply chain resilience [54], educational system resilience [55], and socioeconomic resilience [56,57]. Furthermore, several researchers have considered the relationships and interactions between risk, hazard, uncertainty, and resilience in the era of COVID-19 [58,59,60,61,62,63,64].

**Figure 5:**
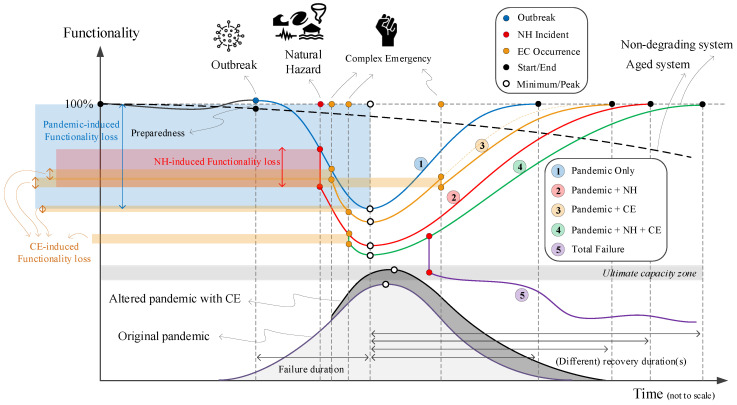
Response and recovery of healthcare system under multi-hazard scenarios during pandemic; Five color lines present the response/recovery of the healthcare system; color (red, blue and yellow) circles show the occurrence of three hazard sources; the black circle shows the start and end point of the resilience curves; the white circle presents the minimum resiliency (or peak pandemic); the light and dark gray bell shapes are the pandemic progress over time in the original form and altered by CE, respectively; and finally, the colored transparent rectangle on the top left side of the figure presents the individual/cumulative functionality loss.

Figure 5 qualitatively illustrates a few potential scenarios (some with rare probability) that could happen within a healthcare system. Again, our primary metric is loss of life, which should be controlled (i.e., reduced) during a pandemic. Similar scenarios can be designed for social and economic aspects, which are ignored in this paper. One may note that combining these three hazard sources with different nature is a challenging task because their spatio-temporal domains are different. While most crises or disasters are constrained within a relatively limited space and time, pandemics persist and reverberate for months or even years [65]. Establishing our three main hazard sources as a NH, pandemic, and CEs, the following five scenarios can be discussed, see Figure 5:

1. Pandemic only: This is a single-hazard scenario and assumes no other concurrent hazard threatens the healthcare system. Based on this figure, the healthcare system is assumed to be initially in either full functionality (i.e., 100%), in a degraded mode (>100%), or in an upgraded mode (<100%). Degraded functionality could be caused by aging facilities or personnel and medical equipment shortages. Alternatively, upgraded functionality could be due to the preparedness of a system with prior knowledge about the possible occurrence and dimensions of such a pandemic [66].
  The performance, or functionality, of a system is reduced with increasing numbers of positive COVID-19 cases. The system has minimum functionality (more or less) when the pandemic is peaking. By reducing the number of infections and designating additional monetary and logistical recourses to the issue, the system recovers from this adverse effect. The transitioning from response to recovery, including consideration of assessment, management, and communication of risk and uncertainty over time was discussed by Menoni and Schwarze [67].
2. Pandemic + Natural Hazard: This is a double-hazard situation that assumes a natural hazard (e.g., earthquake, flood) hits the community during a pandemic. Examples are provided in Section 2.2. The NH-induced functionality loss in this scenario is fairly rapid, compared to the slow reduction of functionality in the pandemic-only case. A natural disaster may impose extra pressure on the healthcare system by occupying a considerable amount of overall hospital capacity. It can also cause a large evacuation, which in turn increases the risk of viral infections among displaced people.
  Following the sudden functionality loss due to NH, the final compound loss of functionality in this scenario is more than in the pandemic-only one, assuming that the natural hazard can turn into a disaster. Recovery in this scenario is also longer because the natural disaster may cause some physical damage to the healthcare system, which would not occur in the pandemic-only scenario.
3. Pandemic + CE: This is also a double-hazard situation in which multiple CEs (e.g., political conflict, protests) occur during a pandemic. Each of these events, depending on their severity, may or may not reduce the functionality of the healthcare system, including reductions in financial resources, global collaborations, and/or data sharing. Compared to the pandemic-only scenario, the recovery time is higher.
  On the other hand, the occurrence of such CEs may impact the original pandemic transmission curve by intensifying its peak and elongating its endurance time, see Figure 5 (transition from light gray to darker one).
4. Pandemic + NH + CE: This is a very low-probability, high-consequence situation in which all three hazard sources occur in a relatively short timeframe, though not necessarily at the same time. Such a scenario might cause the largest functionality loss and longest recovery time. One may recognize some states within the U.S. exposed to such multi-risk by overlapping the three maps in Figure 2.
5. The final scenario is an intense version of any of the previous four scenarios. The healthcare system in each county has a limited capacity (e.g., ICU rooms, ventilator machines), and may fail if the imposed demand becomes higher than the “ultimate capacity” of the system [68]. A potential solution is to flatten the transmission curve by imposing stronger stay-at-home orders.

## 5. Multi-Hazard Evacuation Models

While each of the above-mentioned single hazards may lead to direct fatalities (the main metric discussed in this paper), they may also cause some indirect effects. More specifically, the combination of a pandemic with either NHs or CEs will cause higher infection rates and potentially more fatalities. While mass gatherings due to CEs can be controlled or prevented to some extent, see Section 2.3, evacuations forced by NHs are usually inevitable.

Evacuating a large number of people during a pandemic is challenging, given the public health advice to slow the spread of new infections. As mentioned in Section 2.2, during the 2020 Michigan dam failures, a total of 11,000 people were evacuated. The concept of crowd simulation was already studied in different forms [69,70,71]. While there are multiple models to simulate the evacuation of people during hazards, such as wildfires [72,73], earthquakes [74], and tsunamis [75], very little research can be found that directly addresses this issue during a pandemic or epidemic [76]. Therefore, developing such multi-hazard evacuation models is a missing link towards overall community resilience.

**Figure 6:**
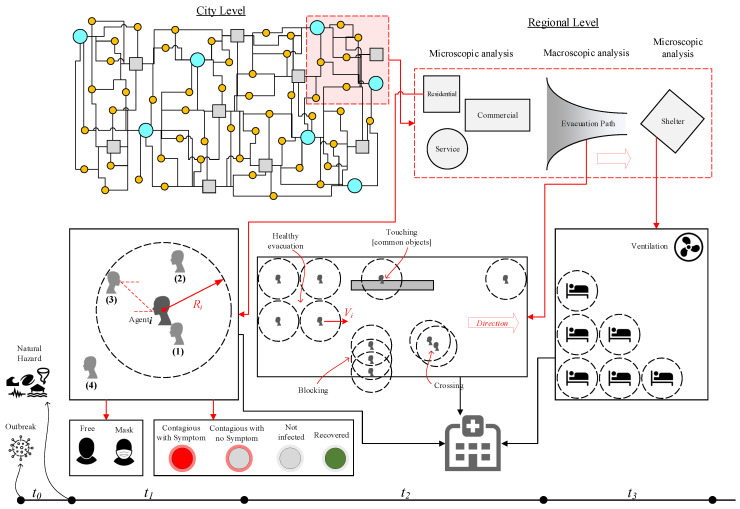
Evacuation and sheltering in pandemic era after a natural hazard; in city level figure (**top left**), the circles and squares present different types of buildings; the timeline axis presents the pandemic era before natural hazard occurrence, $t_{0}$, during staying inside a confined place, $t_{1}$, during evacuation, $t_{2}$, and during sheltering, $t_{3}$.

Figure 6 qualitatively presents a general framework to simulate the evacuations during a concurring pandemic and natural hazard. Any new model should include microscopic and macroscopic crowd models. While the microscopic models take into account spatial-temporal information at the individual-level, city-level evacuations, sheltering, and effective social distancing are governed by macroscopic models. A natural hazard usually hits the entire city (like during an earthquake) or just part of it (like in a flood), forcing people to evacuate with little time. Having an emergency action plan (EAP) is a key factor in responding quickly to hazards, as these plans guide people to the nearest, safest shelters. One may isolate only a small portion of the city (as shown in Figure 6; top right) and develop the model in three parts:

- Confined space crowd models investigate the occupants’ (or agents’) exposure inside a building during a pandemic and right before or after a natural hazard. In this model, various factors, such as the distance between individuals, the type of transmission contact (e.g., airborne, droplets), and time of exposure, should be considered. The uncertainty associated with the spread of disease can be addressed as one of four potential cases shown in Figure 6:
  1. Direct physical contact (e.g., touching).
  2. Within the social distance: the exposure might happen if the agent${}_{j}$ falls within the social distance (about 2.0 m) of agent${}_{i}$.
  3. Being face-to-face within the social distance: the transmission of COVID-19 is higher when the individuals are facing each other or their faces are at a certain angle of each other. This is an important factor especially in commercial or service centers.
  4. Being in the same confined area.
  One may add the following further details to each of four above-mentioned cases:
  - All cases are time-dependent and should be analyzed in the transient mode.
  - All the interactions should be modeled between any two combination of agent${}_{i}$ and agent${}_{j}$.
  - Various constraints should be applied to the simulations including but not limited to: using face covering, contagious with or without symptoms, etc.
- Evacuation models are divided into two parts: evacuating a building and heading towards a shelter. For the latter, factors such as duration, length of travel, difficulty of paths, speed of each individual, potential touching of common surfaces/objects, blocked paths by a group of individuals, and violations of social distancing should be considered.
- Lastly, sheltering is another major concern during a pandemic, and the capacity of shelters should be recalculated to account for safe distancing between individuals, as well as the length of time evacuees will remain there. Among other factors, the functionality of ventilation systems should be managed to avoid potential damage by a natural hazard.
  During all three models, a portion of evacuees might become injured, which should be accounted for in evacuation models and added to the resiliency of the healthcare system, Figure 5.

## 6. Conclusions

This paper highlights the importance and impacts of natural hazards and various complex emergencies in a pandemic era and explains the concept of multi-hazard risk in three hazard scenarios. Two major ideas are qualitatively proposed for future detailed research: (1) the need for resilience models that explore the healthcare system under multi-hazard risk, potential forms of functionality loss, and the recovery duration; and (2) the need for pandemic-specific evacuation and sheltering models that also cover the risks posed by NHs.

While the skeleton of the paper was formed based on the data, hazards, and events that have been reported in the U.S., the idea can be expanded to any other country without loss of generality. While all countries in the world are fighting COVID-19 outbreak, natural hazards (from different types) are also inevitable. For example, since January 2020, several major natural disasters have been reported worldwide, including: (1) 5.3 magnitude earthquake on 22 March in Zagreb, Croatia [77] (one fatality, 27 injured); (2) 5.1 magnitude earthquake on 7 May in Tehran, Iran (two fatalities, 38 injured); (3) earth-fill dam break on 1 May in Uzbekistan (four fatalities, 50 injured, and 70,000 evacuees) [78]; (4) Tropical Storm Amanda, formed on 31 May, along the coast of Guatemala (at least 17 fatalities); (5) Cyclone Harold [79] in the Solomon Islands, Vanuatu, Fiji, and Tonga between 1 and 6 April, which destroyed many homes; (6) wildfire outbreaks in the west, southwest, and south of Iran that burned more than 500 hectares of forest between 27 May and 3 June; (7) multiple floods impacted large tracts of Southern China in June and July due to heavy rains, which affected more than 37 million people, and left about 140 death/missing.

Also, add the ongoing (or new) international complex emergencies to this multi-hazard scenario. The examples of ongoing challenges are: Hong Kong protests, the war/conflicts in the Middle East (such as Syria, Yemen, Afghanistan), etc.

Some other types of complex emergencies are predictable; however, blocking them temporarily may even cause extra future consequences. For example, the authorities in Iran have to face the dilemma of canceling (due to COVID-19) nation-wide university entrance exam early August with about 1.5 million participants (which practically cripple the entire educational system for the upcoming academic year), or risking their lives by a half-a-day exam in the indoor classrooms/environments.

One major conclusion out of this paper is that a multi-hazard situation combining any three hazard sources of pandemic, natural hazard, and complex emergency might have a cascading effect. Since various dimensions of this problem is still unknown (i.e., we do not have a quantitative metric to evaluate the risk, and we clearly are not prepared to face it), the authors implore governments to allocate additional financial resources to multi-hazard risk research, paving the way for a safer, less uncertain future. While the COVID-19 pandemic and all its consequences were unfortunate for the society, some researchers note that it might yield positive impacts for future resilience design, plans, and politics within built environments [65,80]. Last but not least, the author believes that anyone in any position should contribute (to the extent possible) to improve the knowledge related to the COVID-19 outbreak, and as Haas [81] truly said: “*Risk analysts and risk analysis researchers should not be shy about contributing our skills to important policy developments during this crisis.*”

This paper proposed a multi-risk assessment framework in the following general form, *g*, in which the risk increases in the presence of a pandemic, natural hazard, complex emergencies, and in the lack of a sufficient healthcare system:

(6) $$\begin{array}{c} Multi\;Risk∼g\;(\frac{Pandemic,\;Natural\;Hazard,\;Complex\;Emergency}{Healthcare\;System}) \end{array}$$

While this paper proposed a general framework, we did not present a quantitative example (case study). At the time of publication of this paper, the world was in the middle of a COVID-19 pandemic, with no definite database on full interaction of different hazard sources. As a future work, the idea presented in this paper can be applied to the database collected at the national or international levels.
